# Hydrogels for active photonics

**DOI:** 10.1038/s41378-023-00609-w

**Published:** 2024-01-01

**Authors:** Byoungsu Ko, Nara Jeon, Jaekyung Kim, Hyunjung Kang, Junhwa Seong, Suhyeon Yun, Trevon Badloe, Junsuk Rho

**Affiliations:** 1 Department of Mechanical Engineering, Pohang University of Science and Technology (POSTECH), Pohang, 37673 Republic of Korea; 2 Department of Chemical Engineering, Pohang University of Science and Technology (POSTECH), Pohang, 37673 Republic of Korea; 3 Graduate School of Artificial Intelligence, Pohang University of Science and Technology (POSTECH), Pohang, 37673 Republic of Korea; 4 POSCO-POSTECH-RIST Convergence Research Center for Flat Optics and Metaphotonics, Pohang, 37673 Republic of Korea

**Keywords:** Nanophotonics and plasmonics, Micro-optics, Structural properties, Chemistry

## Abstract

Conventional photonic devices exhibit static optical properties that are design-dependent, including the material’s refractive index and geometrical parameters. However, they still possess attractive optical responses for applications and are already exploited in devices across various fields. Hydrogel photonics has emerged as a promising solution in the field of active photonics by providing primarily deformable geometric parameters in response to external stimuli. Over the past few years, various studies have been undertaken to attain stimuli-responsive photonic devices with tunable optical properties. Herein, we focus on the recent advancements in hydrogel-based photonics and micro/nanofabrication techniques for hydrogels. In particular, fabrication techniques for hydrogel photonic devices are categorized into film growth, photolithography (PL), electron-beam lithography (EBL), and nanoimprint lithography (NIL). Furthermore, we provide insights into future directions and prospects for deformable hydrogel photonics, along with their potential practical applications.

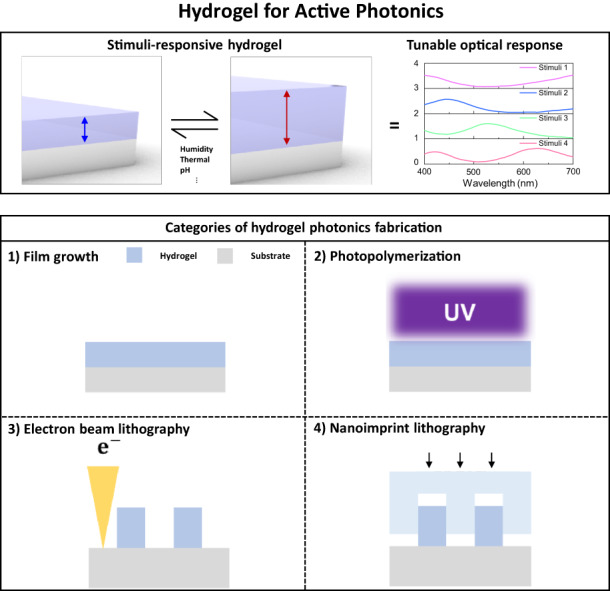

## Introduction

Photonic devices have become essential in our everyday lives, including numerous technologies, from the lenses integrated into our mobile phones to the LiDAR systems utilized in our cars^1,2^. The design of these photonic devices generally involves the utilization of films or structured formats to control over their optical responses. These design approaches enable precise manipulation of light behavior, enabling tailored functionalities and enhanced performance in optical systems. One of the well-known examples of film formats that exploit interference is the Fabry‒Pérot (F-P) interferometer^3,4^. Interference is a fundamental optical phenomenon that arises from two or more light waves interacting with each other, changing their amplitudes and phases. The interference is caused by the reflection and transmission of light at the different refractive index interfaces, where they interact and either reinforce or cancel each other, leading to constructive and destructive interference. Moreover, structured formats provide a higher degree of control over optical properties by incorporating intricate geometries, resonant phenomena, and light-matter interactions^5–9^. Due to the advancement of micro/nanofabrication techniques, such as photolithography (PL)^10^, electron-beam lithography (EBL)^11,12^, and nanoimprint lithography (NIL)^13–15^, it has become possible to fabricate sophisticated structures^16,17^. Structured formats include periodic photonic crystals (PCs) featuring a bandgap, which is a range of wavelengths that cannot propagate through the material owing to their periodic arrangement. Resonance represents another fundamental principle used in structured formats, whereby specific dimensions of a structure with a distinct refractive index interact with light, leading to enhanced absorption or reflection phenomena. Additionally, the concept of scattering in the design of structured formats facilitates enhanced scattering and absorption of light by particles whose sizes are comparable to the wavelength of light (more details are discussed in reference)^18,19^. Furthermore, a new type of photonic device known as a metasurface has emerged, which belongs to the category of structured forms. Metasurfaces use well-designed periodic or quasiperiodic arrays of subwavelength structures that can control the interaction between light and matter^1,6,12,20–22^. Metasurfaces, which consist of nanostructure arrays, have achieved unprecedented performance by enabling user-selective modulation of phase and amplitude in a variety of photonics applications. However, even though the extraordinary optical properties of metasurfaces are determined by the materials and structural design arrangement, there is still a lack of dynamic optical properties after fabrication.

According to a well-known tunable mechanism in photonics, a dynamic optical response in photonics can be attained when one of the following conditions is satisfied^23–29^: (1) control of the incident light^30–38^, (2) modulation of the refractive index of the material or surroundings^39–42^, and (3) control of the geometric parameters of the structures (more details are discussed in this review). Condition (1) typically uses liquid crystals (LC), where the independent LC cells are predominantly attached to the photonic devices for polarization tuning. However, for Condition (2), an approach is used to achieve optical modulation by replacing static materials with dynamic materials (phase change materials^26,39,41–47^, metal oxide^48–51^, etc.) that exhibit differential optical properties in response to external stimuli. For Condition (3), attempts have been reported in deformable materials, wherein their optical response can be controlled by modifying geometrical parameters through external stimuli, such as strain, temperature, humidity, pH, and others. A hydrogel is a three-dimensional crosslinked polymer network produced by the reaction of two or more monomers^52,53^. Hydrogels absorb water and swell in the atmosphere due to the presence of the hydrophilic groups. In particular, the reaction of hydrogels to external stimuli depends on the characteristics of the monomer, charge density, pendant chain, and degree of crosslinking, allowing precise control according to purpose^54–57^. Furthermore, hydrogels have attracted attention due to their optical transparency, biocompatibility, and compatibility with established manufacturing processes, such as coating, self-assembly, and other method. In particular, hydrogels that exhibit both manufacturing compatibility and nontoxicity are preferred in the field of biosensing due to their intuitive sensing through visible changes^58–60^.

In this review, we provide an overview of hydrogels in photonic design and discuss recent progress in hydrogel-based photonics platforms using micro/nanoprocessing techniques (Table 1). In particular, the hydrogel photonic devices are separated based on their formats and include film growth (coating and synthesis), PL, EBL, and NIL; each is described and classified based on their photonics properties and manufacturing processes. Finally, based on an analysis of each, we present a future perspective on hydrogel photonics in terms of fabrication and application.

**Table 1:** Fabrication methods for film/structure-based hydrogel photonic devices

## Properties of hydrogels

The unique properties of hydrogels, with innate deformable properties, cause them to be promising candidates for dynamic photonics applications. In this section, we provide a comprehensive review of the physical and optical properties of hydrogels, specifically focusing on their deformable behavior.

The properties of hydrogels are determined by the interactions between the polymer chains and the water molecules. The polymer chains have hydrophilic groups (–NH_3_, –COOH, –CONH_2_, –CONH–, –OH, etc.), which can interact with water molecules through hydrogen bonding, electrostatic interactions, or van der Waals forces (more details are discussed in reference)^52,61^. These interactions cause the polymer chains to expand and swell, resulting in the characteristic soft and rubbery texture of hydrogels^53^. The deformable behavior of hydrogels can be controlled by adjusting the chemical composition of the polymer chains, the degree of crosslinking, and environmental conditions, such as pH and temperature^62–64^. The deformable mechanism of the stimuli-responsive hydrogels is based on the reversible formation or disruption of crosslinks between the polymer chains^65^. When a stimulus is applied to the hydrogel, the crosslinks can be disrupted or reformed, causing the hydrogel to swell or deswell. For example, temperature-responsive hydrogels, such as poly(N-isopropylacrylamide) (PNIPAAm) hydrogels, can undergo a reversible phase transition from a swollen state to a collapsed state as the temperature is increased above a critical temperature, known as the lower critical solution temperature (LCST)^66^. This phase transition is due to the disruption of the hydrogen bonding between the polymer chains and the water molecules, which leads to the collapse of the hydrogel network. Another example is pH-responsive hydrogels, such as poly(acrylic acid) (PAA) hydrogels, which can undergo a reversible change in their swelling behavior as the pH of the surrounding solution is changed^52^. The mesh size of the polymeric networks can be significantly changed with small changes in pH, and the swelling properties in an acidic or alkaline solution depend on the type of hydrogels. Pendant groups of anionic and cationic hydrogels are ionized above and below the pKa of the polymeric network, respectively. The presence of ions causes a large osmotic force, leading to the swelling of the hydrogel^67^. The other example is electric-responsive hydrogels, such as PAA^68^, poly(2-acrylamido-2-methylpropanesulfonic acid-co-acrylamide) (poly-(AMPS-co-AAm)^69,70^, and poly(2-hydroxyethyl methacrylate) (PHEMA)^71^; these can undergo a reversible change in swelling behavior as an external electric field is applied^72^. When the hydrogels are positioned within an electric field created between two electrodes with an applied voltage, it results in the attraction of charged ions and counterions in opposing directions due to electrophoretic forces. This phenomenon leads to the electroosmotic movement of water molecules, which can cause deformation of the hydrogel. This property has a great advantage in terms of integration with electronics; thus, there has been a growing interest. The deformable behavior of hydrogels can be quantified using several techniques, including gravimetric analysis, swelling kinetics measurements, and microscopy^52^.

Furthermore, the refractive index (*n*) of hydrogels changes according to deformation, which can be exploited for designing stimulus-responsive active photonic devices^52^. *n* of a hydrogel depends on its water (*n* ~ 1.33) content and crosslinking density. When the hydrogel is exposed to a stimulus that triggers swelling, the water content of the hydrogel increases, causing *n* to decrease. Conversely, when the hydrogel is exposed to a stimulus that triggers deswelling, the water content of the hydrogel decreases, causing *n* to increase. These phenomena can be understood through the effective *n*, which considers the composition ratio of the hydrogel and solute. For example, when a pH-responsive hydrogel containing acidic groups is exposed to an alkaline solution, the acidic groups deprotonate, causing the hydrogel to swell. As the hydrogel swells, the water content increases, leading to a decrease in *n*. Similarly, the temperature-responsive hydrogel is exposed to a temperature above its lower critical solution temperature (LCST), and the hydrogel undergoes a phase transition from a swollen to a collapsed state. As the hydrogel collapses, it leads to an increase in *n*. Moreover, hydrogels possess unique properties that respond to external stimuli, causing them to be promising candidates for designing responsive optical devices for a variety of photonic applications.

## Hydrogel-based photonic devices

Hydrogels possess remarkable material properties and stimuli-responsive functionalities; however, to maintain the stimuli-responsiveness of hydrogels after fabrication and achieve desired shapes, compatible fabrication techniques need to be used. In our approach, we classify hydrogels based on the fabrication processes that enable the implementation of film/structure-based photonic devices (Table 1). Devices with a hydrogel film attained by thin film growth skills, such as spin coating and synthesis, can operate as dynamic optical cavities upon exposure to external stimuli. The hydrogel structures fabricated using EBL and NIL can be used as nanocavities, PCs, and meta-atoms, depending on the fabrication methods. Due to the structured hydrogel, expanded dynamic optical responses can be provided. Based on this, recent research trends in hydrogel photonics are introduced in the next section.

### Film format photonic devices

**Fig. 1:**
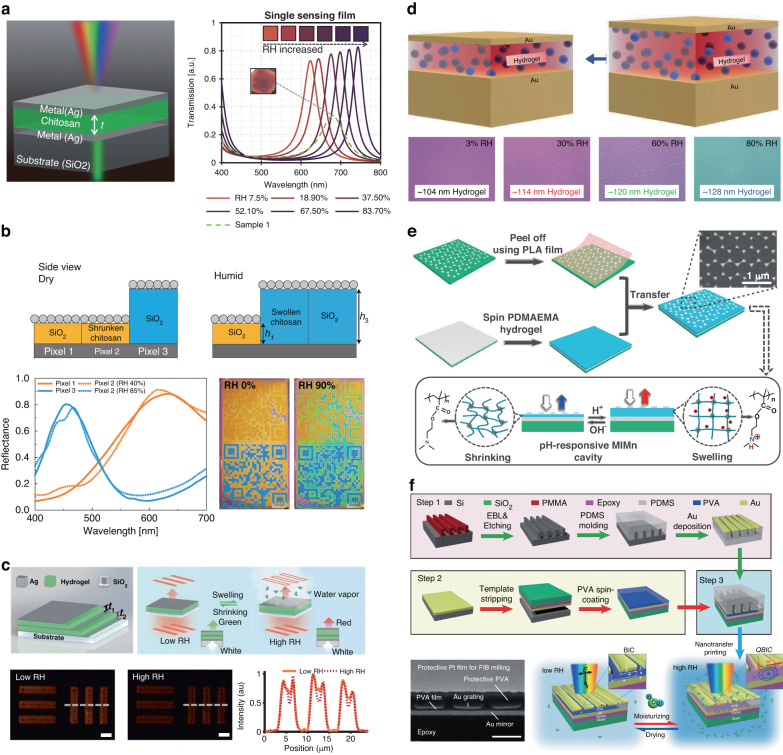
Diverse applications of photonic devices integrated with a two-dimensional hydrogel thin film. **a** Chitosan-integrated metal-hydrogel-metal (MHM) filter and its transmission spectra for various chitosan thicknesses^77^. **b** Humidity-responsive security labels using disordered metal nanoparticles^78^. **c** Multilayered nanoslide for switchable image processing by tuning humidity^80^. **d** Dye-containing MHM cavity for emission tuning^86^. **e** Indirectly built MHM nanoarray plasmonic cavity with pH responsiveness^88^. **f** MHM structure with a transition from the bound state in the continuum (BIC) to the quasibound state in the continuum (qBIC) driven by moisture^90^. Reproduced with permission from Wiley (2020), AAAS (2022), Wiley (2023), ACS Publications (2022), Royal Society of Chemistry (2023), and Wiley (2022)

Extensive research has been conducted on the photonics application of reconfigurable hydrogel films, exploiting the attractive properties of hydrogels. To prepare hydrogel films, precursor solutions containing monomers or uncrosslinked polymers are initially used to form a film, which subsequently undergoes a sol-gel transition to create the desired network structure. A variety of widely employed film-forming techniques, such as spin-coating, dip-coating, solution casting, spray coating, and molecular self-assembly, can be utilized to create films^64^. The simply designed structure, composed of a homogenous hydrogel thin film on a reflective substrate, can be easily fabricated, providing high robustness, fast response, and excellent film uniformity^73^. Different fabrication strategies have been adopted to create hydrogel-based film format photonic devices (Fig. 1). Although there might be differences in the methodology used to form these films depending on the material requirements (e.g., temperature, type of crosslinking), common cases of coating, crosslinking, and synthesis processes are used to create hydrogel thin films. Conventionally, the metal-insulator-metal (MIM) configuration is a powerful and simple solution that can generate a sharp dip and peak^74^. MIM configurations consist of both the reflective mode and the transmissive mode, depending on the thickness of the bottom metallic mirror. In both types of MIM resonators, the properties of the top layer, including its absorption characteristics, influence the optical response of the resonator. When a lossy top layer is used in a transmissive resonator, it absorbs and dissipates energy, resulting in decreased transmission efficiency and weak interference interactions as it exits through the thin (< skin-depth) bottom metallic layer. In contrast, reflective resonators intentionally incorporate a lossy top layer to enhance absorption. In this configuration, incident light is reflected by the thick (> skin-depth) bottom metallic layer, leading to successive interference due to differences in optical path length, which enables the amplification of specific wavelengths^75,76^. The metal-hydrogel-metal (MHM) configuration, in which the insulator layer is replaced with a hydrogel, is intriguing because it can induce tunable optical responses through an external stimulus. From this, a colorimetric sensor was demonstrated by utilizing a chitosan-MHM, which exhibited changes in thickness in response to variations in the surrounding relative humidity (RH). This sensor was successfully integrated with a photovoltaic (PV) cell utilizing a transmissive MHM configuration (Fig. 1a)^77^. The integration of the sensor with the PV cell enabled the generation of an electric signal from transmissive light. The presence of chitosan allowed for the modulation of the transmissive resonance from approximately 600 to 750 nm based on humidity changes (RH 7.5 to 83.7%), thereby enabling tunable coloration. Despite its facile fabrication process, the MHM configuration has proven to be suitable for optical humidity sensors. However, the presence of a densely deposited film obstructed the access of humidity, thereby causing a decline in the responsivity. Consequently, a novel approach has been introduced to enhance sensor responsivity in subsequent research. To improve humidity permeability, the top deposited metal was substituted as the disordered layer of Ag nanoparticles (NPs) in the MHM (Fig. 1b)^78^. While conventional physical deposition methods, such as electron beam deposition and sputtering, were commonly used to create dense and uniform metallic films, they tended to act as barriers hindering the penetration of moisture molecules, thereby reducing responsivity. On the other hand, a film composed of disordered Ag NPs promoted the penetration of moisture molecules. The improved moisture penetration, attributed to the Knudsen effect related to gas flow in the noncontinuum system^79^, contributed to the accelerated responsivity of this colorimetric sensor. The achieved responsivity for each response (RH 20 to 80%) and recovery (RH 80 to 20%) process was 141 ms and 140 ms, respectively. This result demonstrated a significant improvement in sensitivity compared to the previous study, which achieved a response time of 3,800,000 ms. This sensor was used as a humidity-responsive display with applications in anticounterfeiting, providing rapid inspection capabilities by its ultrafast response. Moreover, the upper film, which facilitated optical interactions, caused a response/recovery time delay in stimuli-hydrogel interactions based on its density and material characteristics.

Furthermore, the multilayer film construction for edge-enhanced imaging by stacking metallic and hydrogel was demonstrated (Fig. 1c)^80^. The thermally evaporated Ag and the spin-coated polyvinyl alcohol (PVA) were alternately stacked to form a five-layer nanoslide. When the numerical aperture (NA) increased (from 0 to 0.8), the transmittance trend noticeably decreased (510 to 580 nm). Angular-sensitive transmittance from the multiple interference effect^81,82^ allowed high-frequency pass and edge enhancement at 510 nm. The nanoslide alternatively functioned as a low-frequency-pass filter and bright-field imaging because the multilayer stack had wavelength sensitivity^83^, causing a decrease in transmittance as the incident angle increased to 580 nm. In addition, active optical filtering was achieved by adjusting the RH and switching between bright-field and edge-enhanced imaging due to nanocavity swelling. This had the potential for real-time dynamic image processing, biological imaging, and analog computing.

Moreover, hydrogels can be grown as films via chemical synthesis. Among them, the surface-initiated atomic transfer radical polymerization (SI-ATRP) method is a well-known methodology for growing hydrogel films^84^. SI-ATRP polymerization is initiated by the addition of a monomer, a catalyst, and the SI-ATRP initiator that is attached to the hydrogel matrix. During polymerization, polymer chains are formed through the controlled addition of monomers to the radical sites generated by the catalyst. The polymer chains grow within the hydrogel matrix, resulting in the formation of a hydrogel with controlled chemical composition and configuration. Based on this method, a temperature variation MHM using poly(N-isopropylacrylamide) (PNIPAm), which changed shape at a specific temperature was also reported^85^. Furthermore, photoluminescent hydrogels with added emitters were integrated into the MHM cavity for an emission-tunable platform (Fig. 1d)^86^. The poly(N-isopropylacrylamide)-acrylamidobenzophenone (PNIPAm-BP) containing the emitter, rhodamine B (RhB), was spin-coated on the e-beam-deposited bottom Au layer and cross-linked through exposure to ultraviolet (UV) light. To improve the film quality directly related to the optical properties, an alternative solution was spin-coated in two steps^87^. Subsequently, the top Au layer was thermally evaporated on the hydrogel layer. When the resonance of the MHM cavity aligned with both the absorption and emission bands of the emitter, the emission was optimally enhanced as a result of the synergistic combination of the Purcell factor enhancement and the excitation rate enhancement. The overlapping region with the absorption and emission of an emitter was controlled by humidity to achieve tunable emission because the cavity resonance wavelength could be modified depending on the hydrogel thickness. The sample color was reversibly changed with the increment and decline of the RH value from 3 to 80%, resulting in the redshift of cavity resonance by 40 nm, from 548 to 588 nm and a nearly twofold enhancement in the emission intensity. The significant spectral shift observed was notable and had substantial significance, particularly for sensing applications.

With further advances from the MHM, structural metallic nanoarrays have been used instead of a top metal layer for dynamic plasmonic color displays. The Ag triangle array was detached from the original substrate and transferred onto the spin-coated poly(N,N-dimethylaminoethyl methacrylate) (PDMAEMA) layer by mediating polylactic acid (PLA) film (Fig. 1e)^88^. The Ag triangle nanoarray was selected because of its strong localized surface plasmon resonance properties and simplicity in fabrication using colloidal lithography^89^. This indirect “layer-by-layer” building strategy avoided chemical contact with the hydrogel that tended to occur during direct conventional fabrication for a plasmonic structure. In this plasmonic cavity, gap surface plasmonic resonance and resonance simultaneously occurred, similar to F-P interference. The coupling mode between the top and bottom metallic layers was determined by the thickness of the hydrogel layer. In addition, by changing the pH value of the surrounding environment, the colors of the MHM plasmonic cavity could be precisely controlled because PDMAEMA exhibited a substantial swelling response. A humidity-driven bound state in the continuum (BIC) switch through a transferred metal nanograting on a metal-hydrogel configuration was demonstrated (Fig. 1f)^90^. In particular, the plasmonic BIC was a resonant state of light confined within metallic nanostructures and had a long lifetime, which was advantageous for applications, such as detection and lasing. This device switched from BIC to quasi-BIC (qBIC) under RH exposure conditions. During the RH exposure process, full switching to qBIC was observed due to the increase in resonance intensity along with the shift of the plasmon BIC wavelength, ensuring a response time of less than 1 second. These outstanding optical properties and fast response speeds could be used for applications, such as humidity detection sensors. As mentioned above, the direct transfer of arbitrary structures to hydrogels could expand processable hydrogels.

### Photopolymerization

A photocurable hydrogel precursor composed of a hydrogel and a photoinitiator exhibits the ability to undergo crosslinking under UV exposure conditions^91,92^. The photoinitiator initiates a photochemical reaction upon UV light irradiation, leading to the generation of free radicals. These free radicals facilitate the formation of covalent bonds among polymer chains, thereby establishing a crosslinking network. Consequently, the photocurable hydrogel precursor is capable of forming films and structures when subjected to UV exposure, with or without the use of a mask, owing to the presence of the photoinitiator. Due to the utilization of particles or masks, hydrogel structures can be fabricated at a few hundred nm scale.

**Fig. 2:**
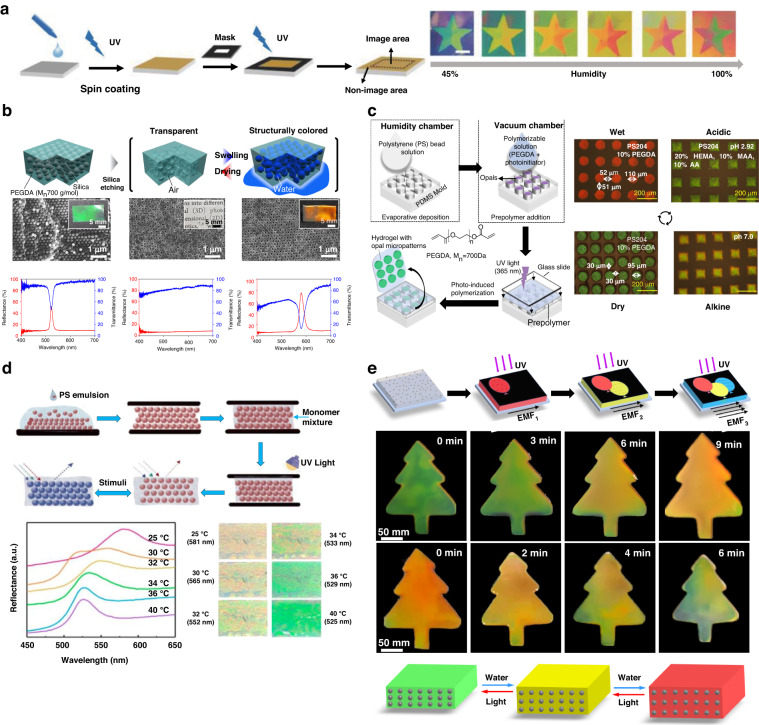
Photopolymerization approaches for achieving hydrogel-based tunable photonic devices. **a** Different structural colors obtained by spatially varying the degree of polymerization using a photomask^93^. **b** Macroporous hydrogel for tuning between the transparent state and iridescent state^97^. **c** Water- and pH-responsive hydrogel composite made of PS beads and PEGDA^98^. **d** Temperature-responsive hydrogel composite made of PS beads and PNIPAm^99^. **e** Magnetic structural color hydrogel composite made of superparamagnetic nanoparticles and PEGDA hydrogel^100^. Reproduced with permission from Wiley (2018), Wiley (2020), ACS Publishing Center (2021), ACS Publishing Center (2022), and ACS Publishing Center (2022)

A colorimetric analysis system based on a simple hydrogel film was demonstrated for the detection of volatile vapors (Fig. 2a)^93^. The poly(HEMA-co-AAc) precursor was coated onto a mirror substrate, exploiting the interference of two reflected light waves at the interfaces of the air-hydrogel and mirror-hydrogel layers. By controlling the degree of polymerization through exposure using a photomask, the film thicknesses were selectively varied during the precursor polymerization process. The resulting hydrogel films exhibited different swelling behaviors in response to the amounts of volatile vapors, leading to highly accurate detection through color changes. Additionally, the exposed surface of the hydrogel enabled rapid response and recovery times by facilitating direct reactions at the interfaces.

The particle or porous structure allows optical modulation via the effective refractive index of the medium or optical path length^94,95^. The enhancement of the light-matter interaction by utilizing PC, which consisted of a particle-/pore-embedded hydrogel, was attempted^96^. In photopolymerization, the dispersion contained the hydrogel, target particle, and photoinitiator. In general, the particle-embedded PC was prepared by a simple process of coating and photocuring a particle-hydrogel dispersion. Additionally, the porous-embedded PC was prepared using a two-step process involving film photocuring of a particle-hydrogel dispersion, followed by the etching of particles.

Various PC-based hydrogel films have been investigated. First, a macroporous poly(ethylene glycol)diacrylate (PEGDA) film demonstrated rapid humidity sensing by reversible switching between a transparent at the dried state and colored at the wet state (Fig. 2b)^97^. This macroporous hydrogel was prepared using a process involving the photocuring of a silica-PEGDA precursor by the selective etching of silica particles. In the dried state, it exhibited high transparency due to the random collapse of the macropores and loss of its ordered arrangement. When the films were swollen by water, ethanol, or a mixture of the two, the macropores were restored and recovered their ordered structure, resulting in structural colors. Notably, the resonance wavelength in the wet state was redshifted with increasing concentration of a minor component in the mixture, which could promise simple and sensitive measurements of trace concentration in mixtures. Moreover, the macroporous hydrogel could be tailored into a size distribution with various shapes, further expanding its applications.

**Fig. 3:**
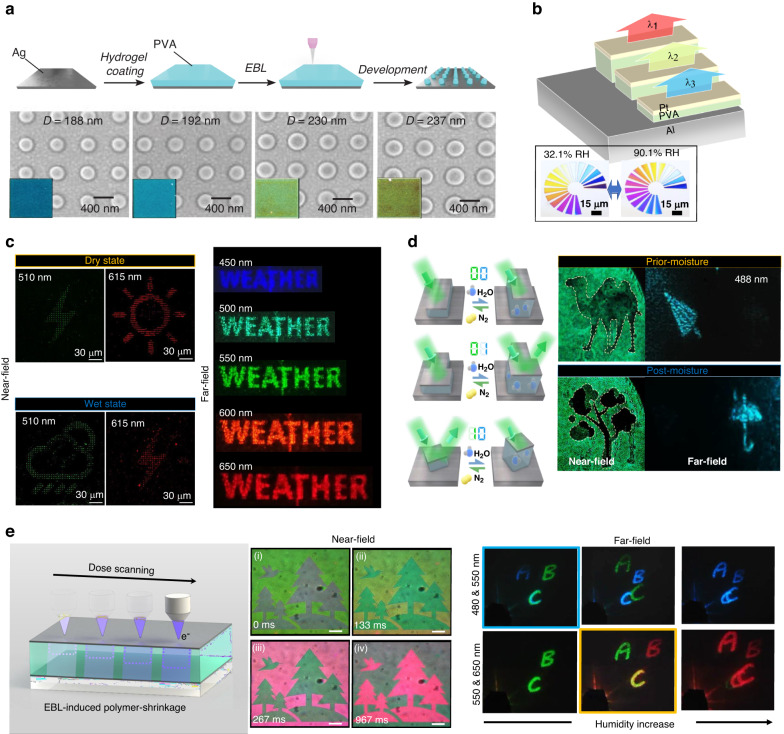
Electron beam lithography approaches for achieving hydrogel-based tunable photonic devices. **a** Direct writing of PVA pillars with continuously varying diameters to enhance surface plasmon polaritons (SPPs)^105^. **b** Reflective-type metal-hydrogel-metal (MHM) structure using humidity-responsive PVA as an insulating layer for full color generation depending on the relative humidity in the visible wavelength^107^. **c** Attainment of nanoprinting in the near field and metaholography in the far field simultaneously by spatially multiplexing the MHM structure to superpixels^108^. **d** Reflectivity change of the MHM structure by tuning between the localized surface plasmon resonance (LSPR) mode and lossy Fabry‒Pérot (F-P) mode for the attainment of nanoprinting and metaholography^110^. **e** Transmissive-type MHM structure using EBL-induced polymer shrinkage for nanoprinting and metaholography^111^. Reproduced with permission from Wiley (2022), Degruyter (2021), Wiley (2021), Wiley (2023), and Wiley (2022)

Furthermore, the micro-opal structured hydrogel has great potential for use in a wide range of sensing applications (Fig. 3c, d)^98,99^. For the rapid and cost-effective process, micromolding-based evaporation-polymerization was demonstrated. The micropatterning procedure began with the evaporative deposition of polystyrene (PS) beads onto patterned polydimethylsiloxane (PDMS). Subsequently, the PEGDA precursor was cast onto the PS bead, and photocuring was initiated to form a bilayered micro-opal structure (Fig. 2c)^98^. The structural color of the fabricated bilayer could be controlled by adjusting the concentration of PEGDA and the size of the beads. Moreover, it could also exhibit pH responsiveness by incorporating acrylic acid (AA) or methacrylic acid (MAA) with carboxylate functionality into the hydrogel. The hydrogel swelling property caused the difference in the spacing between the PS beads according to the RH/pH state, and a significant peak shift of up to 88 nm resulting in distinct color changes was observed. Similar to prior studies, a composite of thermoresponsive PNIPAm hydrogel, PS NPs, and graphene oxide (GO) was introduced to achieve a drug delivery (load/release) system that responded to temperature changes (Fig. 2d)^99^. It was achieved by in situ polymerization of the PNIPAm hydrogel within the gaps of self-assembled PS templates. The addition of GO, with its lamellar structure and light absorption properties, resulted in the construction of structural color templates. The PNIPAm exhibited responsiveness to both temperature and alcohol, which originated from its inherent chemical composition. As the temperature increased over the LCST (~32 °C), the polymer chains within the hydrogel underwent a transition from an extended coil structure to a collapsed globule configuration. This structural change caused the hydrogels to shrink, leading to a reduction in the lattice spacing between the embedded microspheres, which prompted a blueshift of the reflected wavelength, and vice versa. Additionally, when the concentration of the ethanol solution was below 40 wt%, the hydrogel underwent contraction and collapse due to the adsorption of ethanol molecules onto the polymer chains, thereby leading to a reduction in the lattice spacing. Based on these phenomena, real-time monitoring of temperature and alcohol concentration was demonstrated.

Hydrogel composites were created by mixing superparamagnetic Fe_3_O_4_ NPs into a mixture of AA and PEGDA for structural color (Fig. 2e)^100^. Hydrogel-based PC was achieved through magnetically induced self-assembly of the Fe_3_O_4_ NPs, followed by photopolymerization using a mask. The Fe_3_O_4_ NPs were self-organized as PCs by an external magnetic field, resulting in vivid and tunable structural colors within the hydrogels. Notably, the hydrogel-based PC exhibited swelling in water, causing an increase in the gap between the adjacent Fe_3_O_4_ NPs and a noticeable redshift of the reflection peak. Conversely, upon exposure to light, the swelled hydrogels underwent shrinkage due to the photothermal properties of the Fe_3_O_4_ NPs, resulting in a blueshift of the reflection peak as the water within the hydrogel evaporates. Similarly, due to swelling/deswelling, the reflection peak was shifted, which induced a color change. According to this effect, each case showed the redshift or blueshift of the reflection peak observed, ultimately inducing a noticeable color change.

### Electron beam lithography (EBL)

EBL provides an opportunity to fabricate hydrogel structures at the scale of a sub-50 nm^101^. Unlike conventional fabrications for nanostructures that involve etching, hydrogels can directly serve as the resist itself, enabling the direct formation of nanostructures. When an electron beam is exposed to a specific region of the hydrogel film, the high energy breaks the molecular bonds within the hydrogel, forming radicals, which leads to crosslinking of adjacent polymer chains^102,103^. After cross-linking, the structured hydrogels retain their intrinsic characteristics as hydrogels but become insoluble in solvents. This property enables them to be directly utilized as negative resists^102^. Therefore, several studies have been conducted integrated with EBL, which has the advantages of a high degree of freedom and resolution^12^.

By exploiting the simple fabrication and humidity-responsive tunable benefits, a study was proposed to directly fabricate PVA nanopillars by exposing the PVA film to an electron beam^104^. PVA nanopillars whose diameter could continuously change by modulating the exposure energy gradient of the electron beam in the radial direction were fabricated on an Ag film, and the structural color was implemented using the surface plasmon resonance (SPR) phenomenon at the interface between the metal and dielectric. To induce tunable plasmon resonance in the visible regime, a few hundred nm diameter PVA nanopillars were formed via an aligned electron beam onto the Ag mirror substrate, and then a thin Ag film was deposited. As the diameter of the PVA nanopillars increased, the SPR was modulated, inducing tunable coloration and beam steering in the visible regime (Fig. 3a)^105^.

Grayscale electron beam lithography (G-EBL) is a technique that exploits the electron beam-hydrogel crosslinking effect and enables continuous control of the height and diameter of the hydrogel structure through the modulation of the electron beam exposure dose^106^. This technique is attractive because it can provide advanced modulation of complex light corresponding to intensity and phase in the photonics approach. Due to this advantage, many studies have adopted G-EBL to fabricate complex light-modulating nanophotonic devices, such as tunable structural color and holography.

Since hydrogels have a low *n* (~1.5), they exhibit weak modulation performance. To overcome this limitation, attempts have been made to introduce cavity configurations that facilitate modulation through complementary light interference within the cavity.

First, a pixelized MHM was demonstrated, which consisted of a grayscale PVA cavity for humidity-responsive dynamic display (Fig. 3b)^107^. The G-EBL could allow for precise control over the PVA thickness and result in programmable reflective resonance covering the entire visible regime. The initial color was determined by the initial PVA thickness; when the RH exposure increased, the color was redshifted along the PVA swelling. For modulation analysis, the RH condition was modulated within a range of 9.8 to 90.1%, and the resonance shift reached over 50 nm. Due to the combination of G-EBL and hydrogel resists a novel solution was presented to overcome the height limitations in F-P devices.

In addition, photonic devices in a similar configuration demonstrated multiplexing imaging with humidity-responsive behavior based on stepwise MHM (Fig. 3c)^108^. Each MHM pixel was designed with different PVA thicknesses to achieve complete decoupling of amplitude and phase correlation, enabling independent encoding freedom. By employing spatial multiplexing of one cell to the superpixel scheme, RGB triple channels were utilized to independently encode multiple images, such as rainy, lightning, and sunny signs. The use of PVA as the core layer of the MHM resulted in a redshift of the operational wavelength as the RH increased (dry to humid), enabling a real-time dynamic tunable display between interchannels. Consequently, it became possible to simultaneously switch between near-field images and far-field metaholography in real time. As a follow-up research, similar transmission devices were proposed to further advance the field of photonics^109^. This device comprised a photonics system that combined dual-channel dynamic color printing and switchable metaholography. This proposed approach sparked subsequent research in various applications, including tunable displays, encryption, and humidity optical sensor technologies. Furthermore, an independently programmable meta-display switch was demonstrated by encoding meta-pixels into a multiplexed matrix, which included nanoprinting images and metaholography (Fig. 3d)^110^. In this case, it utilized hydrogel swelling dynamics, and the hydrogel-nanoantennas were scaled up or down, actively switching as dominant resonance modes between localized surface plasmon resonance (LSPR) and F-P. This created amplitude-programmable possibilities and encoding degrees of freedom.

A simplified grayscale-MHM fabrication process with the induction of PVA shrinkage using a direct dose scanning process was reported (Fig. 3e)^111^. As mentioned above, the high-energy electron beam was exposed to the hydrogel, and it became crosslinked without a crosslinker, along with volume shrinkage occurring at the same time. By exploiting the volume shrinkage of PVA, the depth of the PVA within MHM could be determined according to the dose scanning, and the development process was omitted, thereby simplifying the fabrication. With the adoption of a similar multiplexing strategy and by encoding the transmission phase into the stepwise MHM, the color image and dynamic projected holographic were modulated in real-time through humid exhalation. The proposed active displays exhibited rapid responsiveness to surrounding RH changes at a millisecond level (<150 ms). Similarly, EBL greatly contributed to the achievement of advanced dynamic photonic devices based on its high process flexibility and resolution.

### Nanoimprint lithography (NIL)

In addition to pattern formation by the light source and electron beam exposure, there have been attempts to fabricate photonic devices with a simple mechanical pressure mechanism of NIL^94,112^. NIL is an intuitive process enabling semipermanent and parallel production by printing onto resin using a soft mold replicated from a master mold. This process can provide higher throughput and larger scalability than other top-down lithography techniques. Thus, the NIL is cost-effective in terms of economics. Another advantage is its high resolution and pattern fidelity. Due to the direct contact between the mold and the substrate, the pattern transfer is highly precise, and the pattern resolutions that are based on mold resolution can be achieved. Moreover, advanced NIL techniques, such as roll-to-roll, are acquiring prominence for their large-scale applicability to a wide range of substrates, including flexible and curved substrates. Recently, imprinting subwavelength metasurfaces by utilizing a bilayer soft mold consisting of rigid hard PDMS (h-PDMS) and flexible buffered PDMS was attempted^113,114^. Due to the high viscosity and low compression modulus of PDMS, which is the main conventional soft mold, it is possible to pattern structures up to 400 nm. Moreover, low-viscosity h-PDMS is able to handle the compression modulus according to the vinyl ratio of the prepolymer and hydrosilane and can be patterned with high aspect ratios and high resolution. Due to this approach, it has become feasible to expand the scope to approximately 50 nm^115^.

**Fig. 4:**
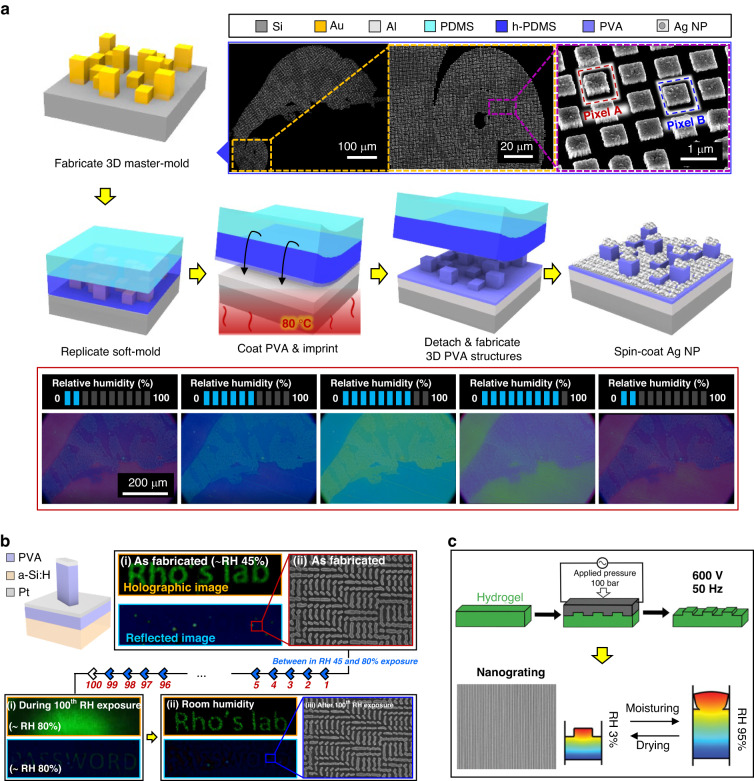
Various nanoimprint lithography (NIL) approaches for achieving hydrogel-based photonic devices. **a** Metal-hydrogel-metal (MHM)-based reflective multilevel nanopixels^116^. **b** Hydrogel metasurfaces for a multiplexed optical encryption system^115^. **c** Ultrafast producible hydrogel nanograting for humidity sensing^117^. Reproduced with permission from Wiley (2023), Nature Publishing Group (2022), and Wiley (2022)

From this, a bilayer soft mold imprinting process was introduced to fabricate nanopixel-based high-resolution displays (Fig. 4a)^116^. Nanopixels (~700 nm^2^) utilized the F-P resonance occurring in the metal-PVA-metal NP structure to display the reflective color. The presence of a hydrogel within the pixel could result in a redshift of the resonance peak by swelling in response to increasing RH 20 to 90%, allowing the entire RGB gamut to be displayed at each pixel. The various thicknesses had different absorption peaks; thus, they could represent different colors for each thickness. This study used an advanced fabrication technique, EBL overlay, to fabricate a multilevel pixelized master mold. A dual-aligned EBL process produced a master mold consisting of nanopixels of different heights (*h*) of Pixel A and Pixel B, which could be replicated in the form of the aforementioned bilayer soft mold. The imprint process could simultaneously transfer not only two height types of pixels but also three level thicknesses, including the residue layers that naturally occurred during the process. Direct spin-coating of Ag NPs onto printed samples enable high-resolution chameleon imaging composed of vivid pixels. The spin coating process that omitted the vacuum process could improve productivity, and furthermore, due to the disordered dispersion of particles, the porosity contributed to the response/recovery time that was based on the Knudsen effect. This colorimetric sensor showed hundreds of ms of a quick reaction similar to a chameleon depending on the RH and had vivid colors and wide color modulation, presenting a potential use as a humidity sensor.

Subsequent research showed that a PVA-based metasurface had irreversible/reversible optical encryption (Fig. 4b)^115^. The PVA metasurface was created by spin-coating aqueous PVA onto a soft mold, and then it was imprinted onto the substrate through the application of pressure. Due to the use of a water-soluble resin, the mold could be washed with water and reused. The device was designed to multiplex holograms (far-field) and structural colors (near-field) with a geometry phase-based design. This device could show independent optical reactions based on the input light source. Additionally, it could exhibit reversible or irreversible properties depending on the deposition process, which in turn enabled the device to be highly flexible and adaptable to its intended purpose as an encryption device. When the hydrogel metasurface was exposed to high humidity conditions, PVA rapidly expanded and destroyed the nanostructures through aggregation between adjacent meta-atoms. In the swelling process, the hidden information (‘PASSWORD’) encrypted in metasurfaces could be decrypted at approximately RH 68%. After 85% RH exposure, the meta-atoms containing hidden information were destroyed, and they could no longer be optical modulators, thus acting as irreversible devices. Another interesting result was that an identically fabricated metasurface could be switched to a reversible device through the deposition of a thin 10 nm layer of metal. Different from the aforementioned irreversible devices, no structural defects were found in the metal-coated PVA metaatoms over 100 times repeated RH exposures. The preservation of the hydrogel metaatom indicated that reversible optical encryption-decryption could be maintained depending on RH exposure. Thus, the tunable morphology and fabrication convenience of hydrogels improved the metasurface applicability, with potential encryption and sensing applications.

The aforementioned research has demonstrated the compatibility between the hydrogel and NIL processes and this has led to subsequent research aimed at ensuring productivity. Thermal-based NIL technology, which enables not only high fidelity but also ultrafast printing, has been utilized for photonic device fabrication^117^. This method can achieve fast production through the integration of the master mold and underneath n-doped silicon-based Joule heaters due to the heat being confined to the structured surface and rapid heating/cooling. A humidity-sensitive reflection grating was fabricated by direct ultrahigh-speed printing onto a hydrogel grown on a substrate using initiated chemical vapor deposition (Fig. 4c)^117^. The hydrogel grating can generate diverse diffraction effects, including structural color, depending on structural parameters and angles. The presence of a hydrogel can promote a gradual color change through an expansion/contraction process in response to changes in humidity conditions, potentially applying to sensors and other devices that can detect changes in humidity. In addition, the newly introduced printing equipment can easily fabricate cm-sized nanostructure arrays at ultrahigh speed (more than 1000 pieces/hour). The development of a rapid fabrication process can facilitate the field of hydrogel-photonics both in terms of commercialization and academic research.

The integration of hydrogels and photonic devices provides an additional means of tuning optical responses through external stimuli and is a unique research field on its own. Additionally, hydrogels are inexpensive, eco-friendly, and compatible with various fabrication processes, causing them to be a promising material. Additionally, their applicability to mass production processes, such as NIL, has been demonstrated, and their potential applications are expected to gradually expand.

## Conclusion and outlook

The development and attainment of hydrogel photonic devices through diverse micro/nanofabrication processes have provided new opportunities in the field of nanophotonics (Table 1). Hydrogels are compatible with diverse micro/nanofabrication platforms, such as coatings, photopolymerization, EBL, and NIL, providing fabrication advantages. In particular, hydrogel-based photonics are suitable for commercialization due to their facile fabrication, such as NIL, enabling mass production. NIL can be combined with roll-to-roll techniques, enhancing the potential for high-throughput fabrication. In addition, hydrogels demonstrate a novel mechanism for geometric modulation based on their inherent swelling/deswelling properties, providing tunability for photonic devices (Table 2). Moreover, the hydrogel stimuli-responsive characteristics are determined by chemical composition, which can be extended to various stimulus-responsive optical devices by replacing the other hydrogel. Continued advancements in the fabrication process and hydrogel development have demonstrated the tremendous potential of the hydrogel photonic devices^92,118–122^.

**Table 2:** Categorization of deformable hydrogel photonic devices

Type	Ref.	Fabrication	Hydrogel	Stimulus	Application
Film	^77^	Spin-coating	chitosan	Relative humidity	Sensing
	^78^		chitosan		Encryption
	^80^		polyvinyl alcohol		Edge-detection
	^84^	Surface-initiate atomic transfer radical polymerization (SI-ATRP)	poly(N-isopropylacrylamide)	Temperature	Sensing
	^86^	Spin-coating	poly(N-isopropylacrylamide)-acrylamidobenzophenone	Relative humidity	
	^88^		poly(N,N′-Dimethylaminoethyl methacrylate)	pH	Display
	^90^		polyvinyl alcohol	Relative humidity	Sensing
Structure	^93^	Photolithography	poly(HEMA-*co*-AAc)	Ethanol	Sensing
	^97^		poly(ethylene glycol)diacrylate	Relative humidity, volatile vapor	Encryption
	^98^	Photo-polymerization	poly(ethylene glycol), poly(HEMA-*co*-AA-*co*-MAA)	Water, pH	Sensing
	^99^		poly(N-isopropylacrylamide)	Temperature	Display
	^100^		acrylamide, poly(ethylene glycol)diacrylate	Water	Display
	^105^	E-beam lithography	polyvinyl alcohol	Relative humidity	Display
	^107^				Display
	^108^				Sensing
	^109^				
	^110^				
	^111^				
	^116^	Nanoimprint lithography	polyvinyl alcohol	Relative humidity	Display
	^115^				Encryption
	^117^		poly(2-hydroxyethyl methacrylate)		Sensing

There have been attempts to commercialize hydrogels with nontoxicity, swelling capabilities, and transparency in bioindustries, such as Lab-on-a-Chip^123,124^ and drug delivery^125,126^. Nevertheless, hydrogels still have not achieved remarkable commercialization in the field of active photonics. Several points need to be considered in the development of future hydrogel-based photonic devices.

In terms of tunable photonics applications, the two main factors of ‘response/recovery time’ and ‘deformation range’ should be used to evaluate the performance of hydrogel photonics. In general, response/recovery time can be explained as the difference in the time required to reach from 10% to 90% (T_10-90_) and 90% to 10% (T_90-10_) intensity of each equilibrium state^78,116^. However, the experimental measurements are not standardized, and a few studies have adopted this method, causing difficulty in comparing the sensitivity. Similarly, the deformation range is similar. The dynamic response of hydrogels occurs by hydrogel molecules, and there is no specific molecular ratio setting for performance comparison between other hydrogels. In particular, humidity-responsive PVA has a nonlinear relationship with the storage modulus of PVA. With an increase in RH, the relaxation process of the polymer is accelerated by the drastic absorption of more water molecules, which leads to an increase in the swelling rate along with the disruption rate of the hydrogen bonds at a high RH^127^. Therefore, setting a specific measurement range is important for verifying the deformation range. Furthermore, the optical characteristics, response speed, and deformation range of the hydrogels can be adjusted to meet the specific requirements of the application. A combination of the hydrogel content and additives of other functional materials (polymers, nanoparticles, stimuli-active materials) can be added to allow for tunability in the optical characteristics, response time, and deformation range.

Additionally, hydrogels can be used to enhance the mechanical stability, reliability, and durability and improve the optical performance. Moreover, through the deeper exploration of the dynamic characteristics of hydrogels at the nanoscale, standardizing the physical properties of hydrogels can promote successful commercialization. However, it remains challenging to analyze the real-time changes in morphology and refractive index associated with hydrogel swelling/deswelling at the nanoscale. Furthermore, the path to commercial success necessitates the establishment of comprehensive performance characterization and testing protocols. These protocols need to assess the reliability and stability performance of hydrogel photonic devices under varying environmental conditions, such as temperature, humidity, or pH. By ensuring their functionality and durability across various conditions, hydrogels can advance the applicability of the hydrogel-based photonic devices.
